# Improving access to rare disease diagnostics in Africa: insights from a multinational pilot study

**DOI:** 10.1186/s13023-026-04202-y

**Published:** 2026-02-05

**Authors:** Albe Carina Swanepoel, Christian Johannes Hendriksz, Renson Mukhwana, Abiola Oduwole, Asmahan T. Abdalla, Emmanuel Ameyaw, Kandi-Catherine Muze, Andrew Auruku, Felix Pinto, Dipesalema Joel, Vesna Aleksovska, Tanya Collin-Histed, Roselyn Odero, Engela Helena Conradie

**Affiliations:** 1 Centre for Human Metabolomics, North‒West University, Potchefstroom, 2531 South Africa; 2 The Desmond Tutu School of Medicine, Faculty of Health Sciences, North‒West University, Potchefstroom, South Africa; 3 A Rare Cause, England and Wales registered Charity, nr:1198461, 6 The Green, Ilkley, LS29 7FF UK; 4 Gertrude’s Children’s Hospital, Nairobi, 00100 Kenya; 5 College of Medicine, University of Lagos/Lagos University Teaching Hospital, Lagos, 100005 Nigeria; 6 Department of Paediatrics, International University of Africa, Khartoum, 12223 Sudan; 7 Department of Child Health, Komfo Anokye Teaching Hospital, Adum-Kumasi, 1934 Ghana; 8 Muhimbili National Hospital, Dar es Salaam, Tanzania; 9 Youth and Women for Opportunities Uganda, Teso region, Eastern, Uganda; 10 Faculty of Medicine UEM, Maputo Central Hospital, Maputo, 1100 Mozambique; 11 Department of Paediatrics and Adolescent Health, University of Botswana/Sir Ketumile Masire Teaching Hospital, Gaborone, Botswana; 12 International Gaucher Alliance, London, EC2A 4NE UK

**Keywords:** Rare disease, Lysosomal storage disorder, Africa, Diagnosis, Dried blood spot testing, Gaucher, Mucopolysaccharidosis, Clinician education

## Abstract

**Background:**

Rare diseases (RDs) in Africa face challenges such as limited diagnosis, expertise, and treatment. FYMCA Medical Ltd., the International Gaucher Alliance (IGA) and the Centre for Human Metabolomics (CHM) at North‒West University (NWU) launched a pilot study in 2022 to create an African RD diagnosis network, focusing on training clinicians and testing dried blood spot (DBS) samples for 6 lysosomal storage disorders (LSDs). The pilot study spanned eight sub-Saharan African countries (Nigeria, Ghana, Tanzania, Kenya, Mozambique, Uganda, Botswana, and Sudan), with samples sent to South Africa for analysis. Clinician training at the CHM was well received, with positive feedback suggesting hybrid learning for future sessions.

**Results:**

The project registered 56 samples in 27 months; Gaucher disease was most common. MPS II was the most prevalent MPS condition, with a prevalence resembling Eastern hemisphere patterns. Genetic variants were consistent with previous studies, but Fabry diagnoses were rare, which indicates the need for more focused programs.

**Conclusions:**

The high diagnostic yield for LSDs highlights the importance of targeted training and DBS cards for genetic testing. Challenges included sample integrity, referral gaps, and logistical delays. Addressing these barriers and fostering ongoing clinician engagement will enhance diagnostic capacity, whereas presenting findings at an African symposium will increase awareness.

**Supplementary Information:**

The online version contains supplementary material available at 10.1186/s13023-026-04202-y.

## Background

Rare diseases (RDs) are currently not a priority in Africa, where the focus remains on communicable diseases such as HIV, malaria, and TB. Challenges include limited government support, lack of clinician expertise, poor diagnostic access, stigma, and poverty. Patients often face delayed or no diagnosis, and few treatments are available or affordable. Public health policies rarely address RDs, despite their growing burden alongside noncommunicable diseases [1].

FYMCA Medical Ltd. is a medical education company dedicated to providing education on RDs and raising awareness in low- and middle-income countries. To date, they have trained approximately 300 clinicians, who have subsequently diagnosed more than a thousand cases of RD. Through this initiative, the lives of some of the most vulnerable individuals in these societies have been positively impacted. The International Gaucher Alliance (IGA) is an international umbrella group representing the interest of Gaucher patients and those of nonfor-profit Gaucher patient groups as well as RD groups throughout the world. The Centre for Human Metabolomics (CHM), housed at North‒West University (NWU, Potchefstroom, South Africa), is committed to delivering innovative, state-of-the-art analytical services to the healthcare sector through advanced technology and a passion for excellence.

In June 2021, FYMCA Medical Ltd. and the IGA invited health care practitioners from Brazil, India, the United Kingdom, and Botswana to join an advisory board working group to support the “Building Capacity in Africa Project”. This group collaborated with 21 representatives, including laboratory personnel, patient organization representatives and clinicians from 9 sub-Saharan African countries, as well as representatives from the UK, Finland, Brazil, and India. Together, they participated in a 14-day virtual advisory board meeting.

The advisory board shared practical insights and highlighted the disparity in services for RD patients, particularly when African countries were compared with high-income countries. During the discussions, “education at all levels” emerged as the most significant barrier in patient diagnosis. Consequently, an educational program that also appeals to policymakers and healthcare funders is essential for advancing RD services in sub-Saharan Africa.

In November of that same year, the organisations organized a virtual educational event focused on “The Obstacles in Diagnosing Patients with suspected Rare Diseases”. The event was attended by 109 delegates from 19 countries, including representatives from 15 African nations.

From these events, the organisations in collaboration with the CHM initiated a pilot study in April 2022 with the aim of establishing an African network for RD diagnosis. In addition, the pilot study determines whether sample collection, transport, analysis, and result reporting can be achieved efficiently and effectively across political borders and over vast distances.

This pilot study comprises two components, namely, training and diagnostics. The training phase was essential for equipping clinicians with the necessary knowledge and skills to identify patients who might be affected by one of the 6 conditions detectable by the CHM’s 6-plex lysosomal storage disorder (LSD) screening test using dried blood spot (DBS) samples. This preparation was required before initiating the diagnostic procedures.

## Methods

### Ethics approval

The study was conducted in accordance with the Declaration of Helsinki and was approved by the Human Research Ethics Committee (HREC) of NWU (NWU-00192–23-A1) on July 24th, 2024. The NWU HREC determined that this research involved minimal risk and approved a waiver for informed consent since secondary data obtained from the diagnostic workflow were used to report descriptive findings. 

### Geographical scope and logistics

Study sites were established in Western Africa (Nigeria, Ghana), Eastern Africa (Tanzania, Kenya, Mozambique, Uganda) and Southern Africa (Botswana), leveraging the established logistics and infrastructure from private pathology groups, as well as existing networks with clinicians in the field of RDs. Additionally, Sudan (northern Africa) was included to compare cross-border transport and logistics between countries with established pathology laboratory network coverage that is linked to South Africa (as in the seven countries mentioned) and those relying on private courier services (as in Sudan). Consequently, this pilot study was conducted across eight countries in Sub-Saharan Africa, all of which referred collected samples back to South Africa; the pilot study had two components, namely, “Training” and “Diagnostics”.

### Training program

Primary care physicians identified for the pilot screening project attended a one-week, fully funded, in-person training program that was presented at the CHM from 30 May to 3 June 2022.

The aim of the training was to equip the clinicians with the required knowledge and skills to identify patients suspected to be affected by one of the 6 conditions that can be tested with the 6-Plex LSD test.

LSDs are rare, complex conditions, the majority of which are caused by the intracellular accumulation of macromolecules due to the deficiency of a specific lysosomal enzyme. These disorders are characterized by a broad range of clinical manifestations, depending on the specific substrate and the sites of accumulation, which vary according to the LSD type. They are typically progressive, life-limiting and life-threatening in severe cases. [2] More than 60 different LSDs have been identified to date [3], with an estimated overall prevalence of 1 in 7000–8000 live births [4, 5].

Excluding other general topics related to inherited metabolic diseases (IMDs) and conditions that should be considered in the differential diagnosis of these patients is not recommended. Therefore, the program addressed the 6 conditions within a broader context of IMDs.

The program commenced with a general overview of the IMDs and the methods and resources that support the diagnostic process. This was followed by a detailed overview of the 6-plex LSD test and the typical presentations of the 6 conditions it screens for. Finally, selected common presentations were explored in depth, incorporating other rare diseases and more common conditions. Throughout the course, participants engaged with case studies that required them to apply the resources presented to solve diagnostic challenges.

The training program was considered completed once all participants had received a certificate of attendance, which was presented to the attending clinician if he/she had attended 100% of the program and successfully completed all the case studies.

### Diagnostics

#### Informed consent

The 6-plex LSD was developed and implemented for diagnostic purposes. Any South African or African patient (regardless of age, sex, or race on any other demographic identifier) could be referred for testing by the physician being consulted.

The physicians and patients were provided with information on LSDs, a diagnostic consent form, and clinical information, as well as instructions on how to collect the sample and the DBS card, which were packed together in a sample collection kit. All the completed forms were sent to the CHM together with the collected sample.

#### Sample collection and transportation

The participating primary care physicians were each provided with testing kits for 20 patients suspected of suffering from one of the six LSDs, following the instructions given during the training session.

Each kit included the following: (i) pair of sterile gloves, (ii) BD Microtainer Quickheel Lancet, (iii) Whatmann 903 DBS card, (iv) alcohol swab, (v) DBS sample collection instructions, (vi) patient information form, (vii) consent forms, and (viii) clinical information form.

Given that specimen quality significantly influences the sensitivity within a bioanalytical workflow [6], DBS cards were selected for this pilot study. DBS cards provide a cost-effective and simplified approach to blood specimen collection, preservation, and transportation, particularly in hard-to-reach populations [7]. Additionally, they offer a reliable, robust and practical alternative to venous blood for genetic testing [8].

Individuals were eligible if the referring physician suspected one of the six LSDs screened by the 6-plex assay based on recognised clinical features such as hepatosplenomegaly, developmental delay, dysmorphism, abnormal dentition, neuropathy or skeletal abnormalities, as expanded on during the in-person training session.

Patients were included if a complete DBS card and accompanying consent and clinical information forms were received. Samples were excluded if blood spots were insufficient, oversaturated, contaminated, or accompanied by incomplete documentation.

After collection, the samples were sent to a specific local pathology laboratory in each of the participating countries. The CHM arranged with these labs to transport the samples back to South Africa. As part of the pilot study, participating countries were offered screening for the most common lysosomal storage disorders. A 6-plex assay was performed on each DBS sample to test for Gaucher, Pompe, Fabry, MPS, ASMD and Krabbe diseases.

Upon receipt at the CHM, the samples were assigned a unique requisition number (identifiable with a barcode) and logged onto Skylims, the laboratory information system in use at the CHM, to be included in the routine diagnostic analysis workflow.

#### Analysis

The CHM’s 6-plex LSD screening test method makes use of a kit-based assay (utilizing PerkinElmer’s NeoLSD^TM^ MSMS kit) with brand-specific adjustments: (i) an Agilent 6470 Triple Quadrupole (TQ) LC‒MS/MS, equipped with a JetStream electrospray ionization (ESI) source operated in positive ion mode; (ii) chromatographic separation on a C18 column (2.1 × 55 mm, 1.8 µm) using a gradient at a flow rate of 0.4 mL/min; (iii) dynamic multiple reaction monitoring over an 8 min runtime. The method was validated for diagnostic use according to the ISO 15189 validation guidelines. Internal quality control for the LC-MS/MS assay included kit-provided positive and normal controls in every batch, which were required to meet lot-specific acceptance limits. For external quality control, the laboratory participates in the Centers for Disease Control and Prevention (CDC) laboratory quality assurance program. Routine system checks—such as system and extraction blanks—were incorporated to monitor background, carryover, and extraction performance, ensuring reliable analytical results.

The enzyme activities were evaluated by measuring specific products generated when enzymes reacted with substrate analogues (part of the kit), and these enzyme activities were expressed as µmol/L/h.

The CHM’s 6-plex LSD test is intended for the quantitative measurement of 6 individual LSD-associated enzymes, namely, (i) acid sphingomyelinase (ASM) and (ii) beta-glucocerebrosidase (ABG), also known as acid beta-glucosidase. (iii) Acid alpha-glucosidase (GAA), (iv) alpha-galactosidase (GLA), (v) galactocerebrosidase (GALC), and (vi) alpha-L-iduronidase (IDUA).

The 6-plex LSD test enables clinicians to screen patients with a clinical presentation indicative of one of the following 6 LSDs: (i) Gaucher disease, suggested by low calculated ABG activity; (ii) Fabry disease, suggested by low calculated GLA enzyme activity; (iii) Pompe disease, suggested by low calculated GAA enzyme activity; (iv) Krabbe disease, suggested by low calculated GALC enzyme activity; (v) Mucopolysaccharidosis type I (MPS I), suggested by low calculated IDUA enzyme activity; and (vi) Acid Sphingomyelinase Deficiency (ASMD), suggested by low calculated ASM enzyme activity.

**Fig. 1:**
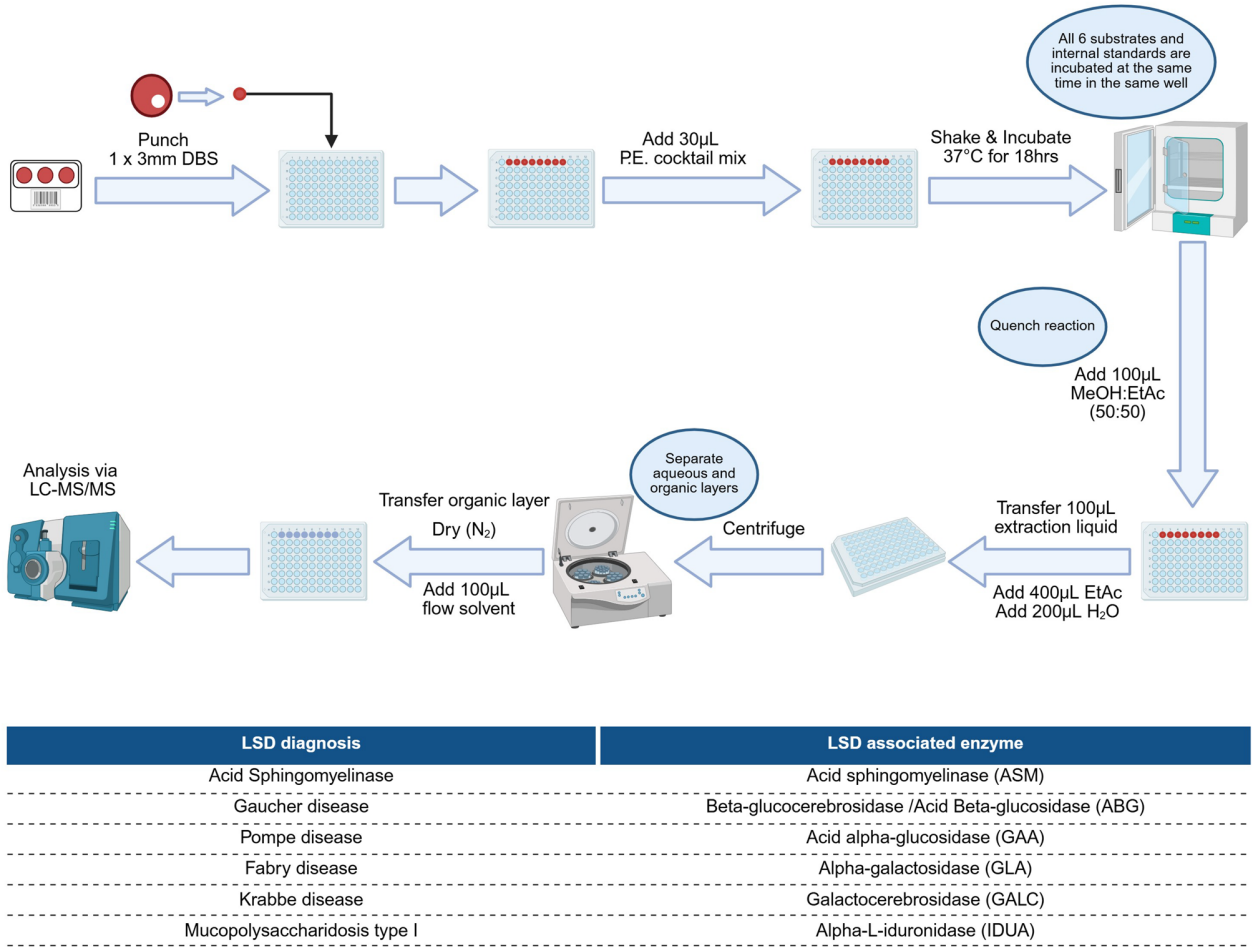
Workflow of the CHM’s 6-plex LSD screening method from a single DBS for six LSDs. Created in https://BioRender.com

Figure 1 provides a graphical summary of the analytical method SOP and the 6 LSDs tested with their corresponding enzymes.

Any results with positive enzymatic analysis were followed up with genetic confirmation. In addition, lyso-GL1 and lyso-GL3 were also measured in instances where Gaucher disease and Fabry disease, respectively, were suspected. Lyso-GL3 is routinely performed when Fabry disease is suspected in a female patient, followed by genetic testing if appropriate. Similarly, expanded enzyme analysis was performed in all instances where alpha-iduronidase levels were normal, but MPS was suspected clinically, followed by genetic testing if needed. All the results were reported via Skylims, with automatic routing to the referring clinician via email. A video conference was arranged in all instances where a rare disease was diagnosed via an online platform to discuss further management of the patient and to arrange for biobanking of samples, if so desired by the patient and/or guardians.

## Results

### Training

The in-person training session was effective overall and well received, as shown in the score in Supplementary Figure 1 (Additional file). The participants provided positive feedback and suggested hybrid learning options as well as including a presentation slot for participants to bring a case from their practice to discussions to ensure that all participants contributed.

The specialties of the attendees included 2 paediatricians, 4 paediatric endocrinologists and 1 clinical pathologist specializing in medical genetics. Two attendees shared their knowledge of their in-person training with clinicians at their hospital to also include those clinicians’ patients in this project. The attendee from Botswana included 5 additional clinicians, and the attendee from Nigeria included 1 additional clinician.

### Diagnostics

The results included in this publication are from participants recruited from April 2022 to June 2024. The project is still ongoing. The total number of participants recruited over this period (*n* = 56) had a median age of 4.5 years (IQR = 9) and had a greater percentage of male participants (*n* = 34, 60.7%). The participants diagnosed with an LSD during this period (*n* = 23) had a median age of 5 years (IQR = 8.5), with an almost equal distribution of male (*n* = 12, 52.2%) and female (*n* = 11, 47.8%) participants.

**Fig. 2:**
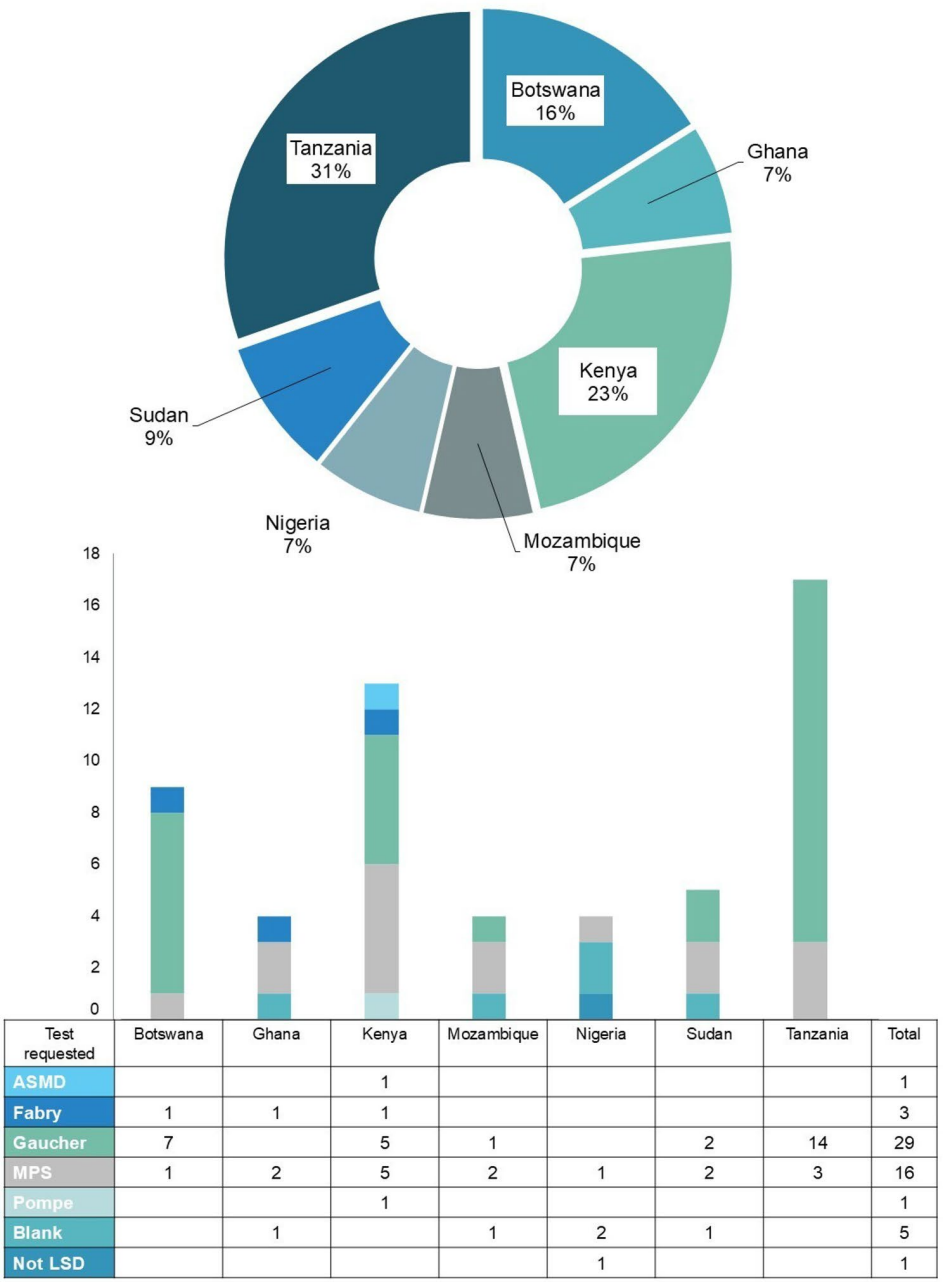
Percentage referrals per country (pie chart) and breakdown of tests requested per country (bar graph)

Figure 2 provides a breakdown of referrals per country as well as the requested tests per country.

Tanzania (17 samples, 31% contribution to total), Kenya (13 samples, 23% contribution to total) and Botswana (9 samples, 16% contribution in total) have made the most referrals thus far, with Sudan referring 5 samples (9% contribution to total) and Ghana, Nigeria and Mozambique referring 4 samples each (relating to a 7% contribution to total for each). No samples have been received from Uganda.

All countries requested at least 1 MPS test, with 16 tests requested in total. Gaucher was the most requested test (*n* = 29) but were not requested by all the participating countries. Some forms were left blank, with no indication of the suspected LSD, and 1 form requested testing for a condition that is not one of the 6 LSDs tested for this project.

Table 1 shows a summary of the LSDs tested with the 6-plex LSD kit, their global incidence and prevalence according to Orphanet and the number of diagnoses per LSD tested in this study. For this pilot study, diagnoses were made for MPS, Gaucher and Fabry but not for ASMD, Pompe or Krabbe.

**Table 1:** Lsds tested with the 6-plex LSD kit, global incidence and prevalence and diagnosis in this study

LSD	Global incidence	Global prevalence	Numbers and percentages for pilot-study Total number of tests(*n* = 56)
Fabry (Orpha:324)	7 in 100,000	1–5/10 000	1 (1.79%)
Gaucher (Orpha:355)	1 in 100,000	1–9/100 000	12 (21.4%)
MPS I (Orpha: 579)	1 in 100,000	1–9/1 000000	3 (5.36%)
MPS II (Orpha: 580)	2 in 100,000	1–9/1 000000	6 (10.7%)
MPS IVa (Orpha:309297)	15 in 100,000	1–5/10 000	1 (1.79%)
Niemann-Pick A/B (Orpha:618891)	Not specified	< 1/1 000000	0
Pompe (Orpha:365)	Not specified	Unknown	0
Krabbe (Orpha:487)	0,4 - 1 in 100,000	1–9/100 000	0

**Fig. 3:**
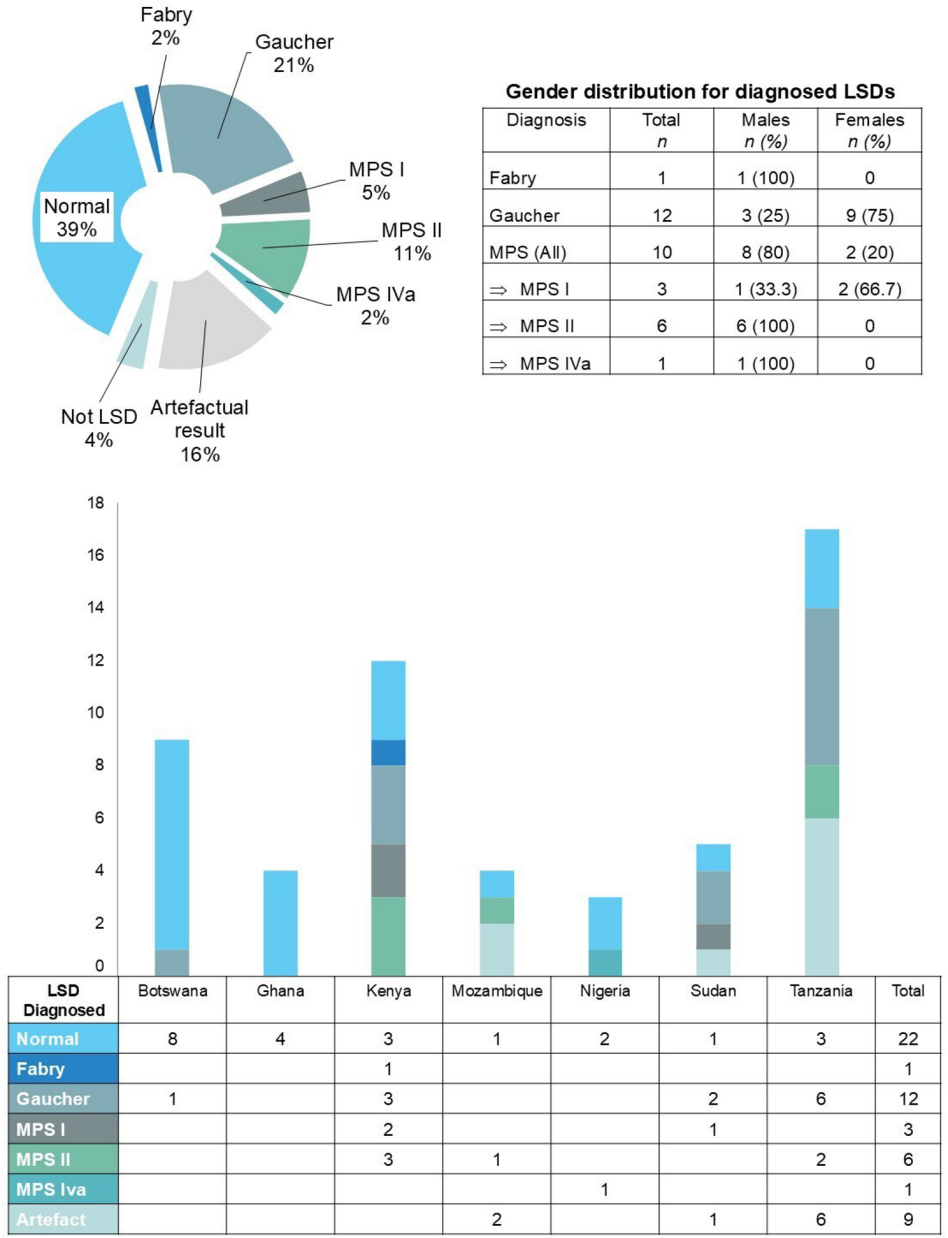
Pie-chart of overall diagnoses, gender distribution and diagnoses for each participating country

Figure 3 shows the pie chart of the overall diagnoses for all participants, the sex distribution of the positive LSD diagnoses and the type and number of diagnoses per participating country. With respect to overall diagnosis, 22 of the referred samples had a normal test result (39% of the total number of referrals), and 9 of the referred samples showed an artifactual result (16% of the total number of referrals). We confirmed 12 cases of Gaucher, 1 case of Fabry and 10 cases of MPS (3 cases of MPS I, 6 cases of MPS II and 1 case of MPS IVa), as shown in Table 1. Two samples that were referred were not LSDs; one presented with clinical CDG/Zellweger (from Kenya), and the other presented with known Alkaptonuria (from Nigeria); these two cases are not included in the bar graph.

The single Fabry diagnosis was for a male participant; the diagnosed Gaucher participants were mostly female (*n* = 9, 75%), whereas the MPS-diagnosed participants were mainly males (*n* = 8, 80%), with only MPS I being diagnosed in 2 females.

When considering the diagnoses made per participating country, most of the samples having an artefactual result came from Tanzania, whereas all countries had samples that showed a normal result. Kenya had the most MPS diagnoses (5 samples), whereas Mozambique, Nigeria and Sudan each had 1 positive MPS diagnosis. Tanzania had the most Gaucher diagnoses (6 samples); Kenya, Sudan and Botswana had 3, 2 and 1 positive Gaucher diagnoses, respectively.

**Fig. 4:**
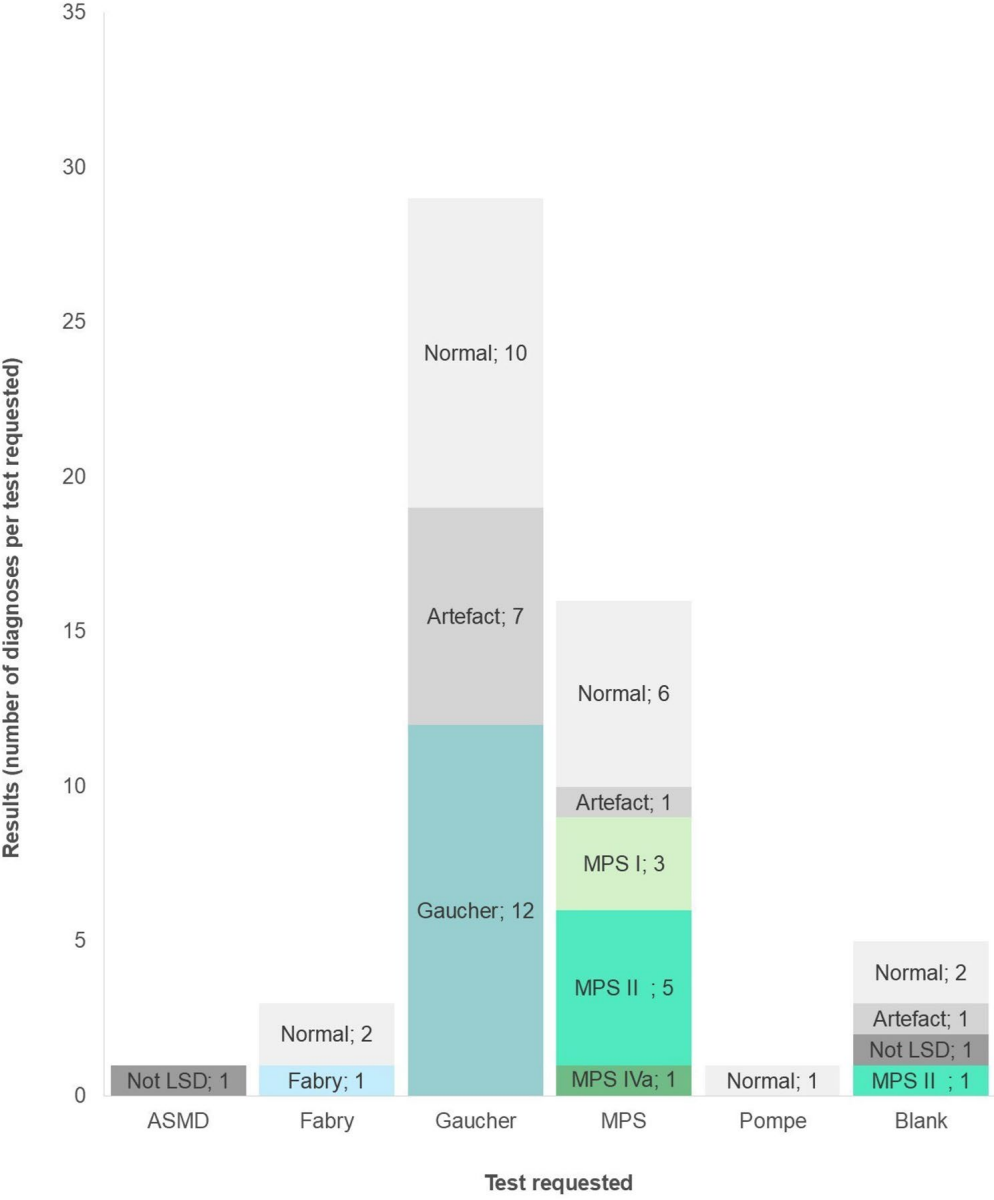
Comparing the requested test to the positive diagnoses

Figure 4 shows the percentage of diagnoses for each requested test. The x-axis indicates the requested tests, whereas the bars indicate the number and percentages (as shown on the left) of positive diagnoses for each condition along with normal, non-LSD and artefactual results. This graph shows that 30% (*n* = 1) of the requested Fabry tests had a positive Fabry diagnosis; 41% (*n* = 12) of the requested Gaucher tests had a positive Gaucher diagnosis; 56% (*n* = 9) of the requested MPS tests had a positive MPS diagnosis; and no ASMD or Pompe requests had a positive diagnosis. No Krabbe requests were made by any of the clinicians. In 20% (*n* = 1) of the cases where a specific test was not requested, a positive MPS diagnosis was made. The 1 form that requested testing for a condition that is not one of the 6 LSDs tested for this project was not included in Fig. 4.

**Fig. 5:**
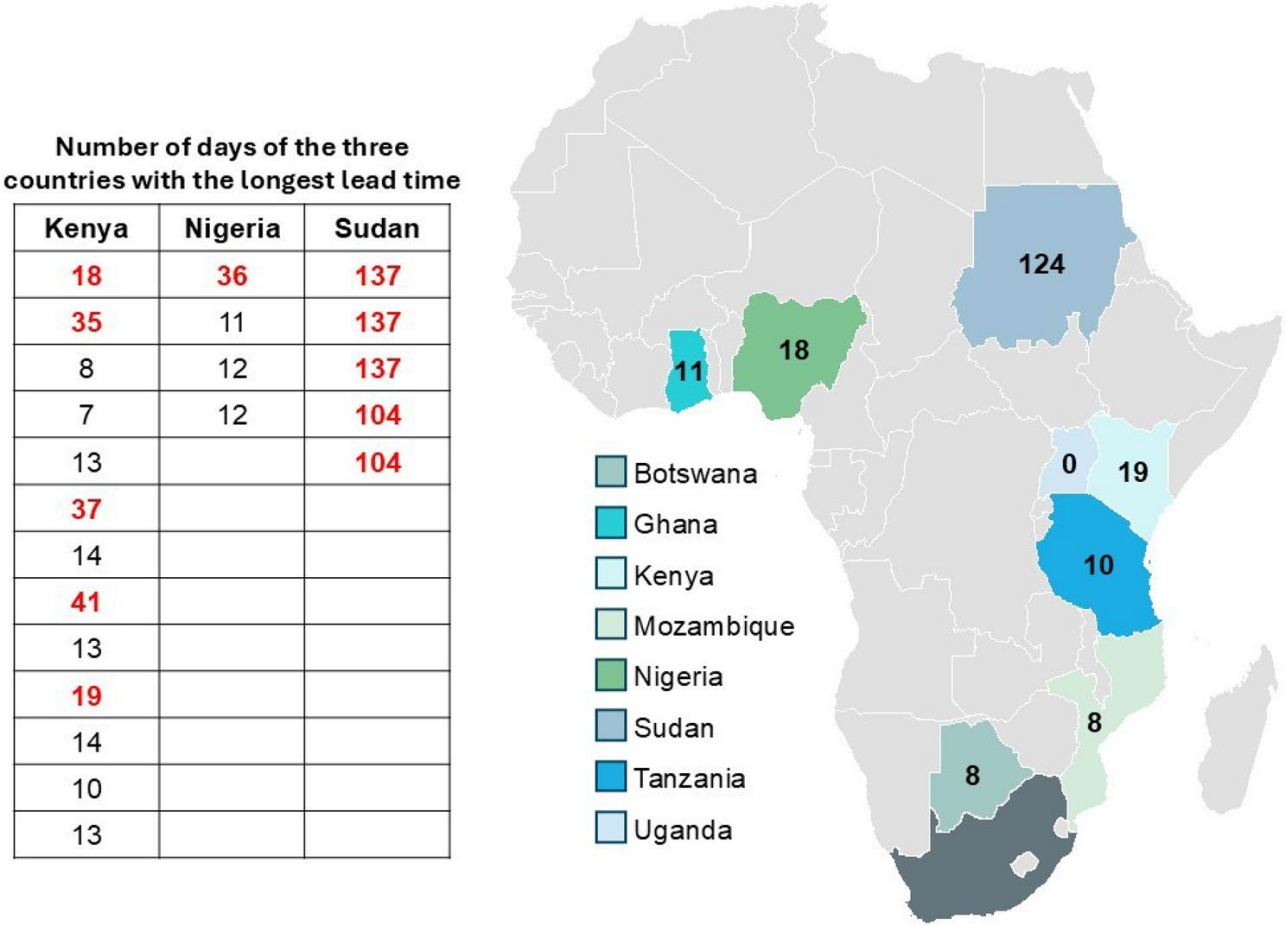
Average number of days until registered per country

**Fig. 6:**
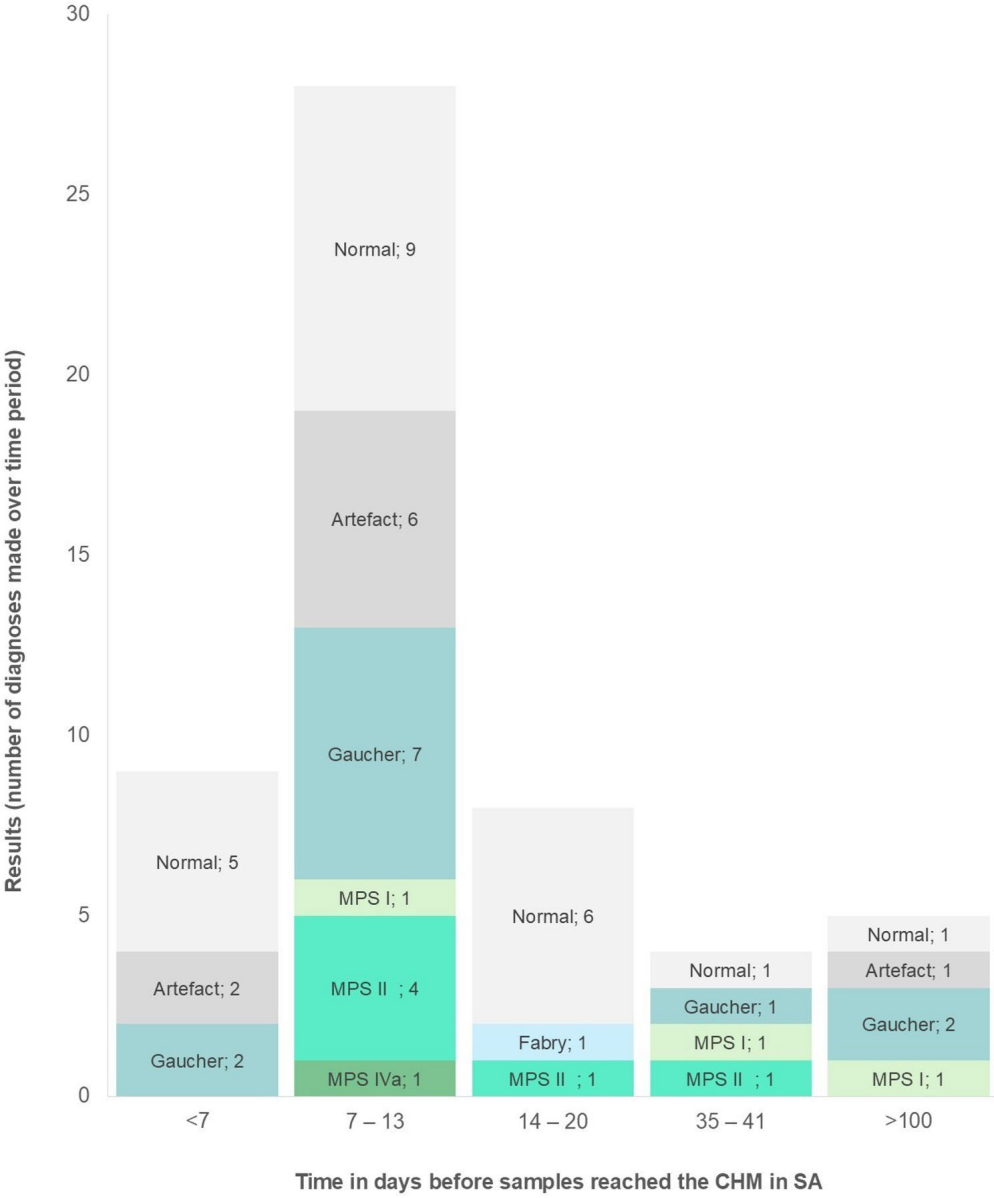
Comparing results for number of days until samples reached and were registered at the CHM

Figure 5 provides a layout of the average number of days until samples were registered at the CHM, SA, according to each participating country. Mozambique and Botswana, both neighbouring countries of South Africa, had the shortest average number of days before registration of 8 days; the average number of days to registration for Ghana and Tanzania was 11 and 10, respectively. For the three countries with the longest lead times until registration, a table is shown in Fig. 6, which shows the number of days for each sample referred by that country. Kenya showed an average of 19 days until registration; most of the samples had a much shorter time period before registration, comparable to that of its neighbouring country, Tanzania (average of 10 days); Kenya had only 5 cases identified as having delayed transport time (as indicated in red). In the same way, most of Nigeria’s samples reached the laboratory within 11 or 12 days (similar to that of Ghana), but the average of 18 days is attributed to 1 case of delayed transport (as indicated in red, taking 36 days). Sudan, which does not form part of the established pathology network, had an average of 124 days, with all cases exceeding 100 days until registration from the time of sample collection.

Figure 6 shows the number of diagnoses made according to the time it took samples to reach and get registered at the CHM in SA. The greatest number of artefactual results were of samples that reached the CHM within 7–13 days. The 5 samples that were referred from Sudan, which all exceeded 100 days from the time of sampling until registration at the CHM in SA, had only 1 artefactual result, while 3 positive LSD diagnoses were made. This shows that the number of days taken to register the samples did not influence the quality of the sample.

All referred samples with a positive enzymatic result were followed up with genetic confirmation. The presenting clinical features, that led to the requested testing, diagnoses and genetic variants, are summarized in Table 2.

**Table 2:** Variants linked to the pilot study diagnosis

Presenting features leading to testing request	Diagnosis	Variant	Variant 2
Cascade testing grandfather confirmed Fabry	Fabry	c.644A > G (p.(Asn215Ser))	
Hepatosplenomegaly, Failure to thrive	Gaucher	c.1448T > C (p.(Leu483Pro))	Not detected but biochemically confirmed
Developmental delay, hepatosplenomegaly	Gaucher	c.1448T > C;1483 G > C;1497 G > C	c.1246 G > A (p.(Gly416Ser))
Hepatosplenomegaly, failure to thrive, Anaemia	Gaucher	c.1448T > C;1483 G > C;1497 G > C	c.1504C > T (p.(Arg502Cys))
Hepatosplenomegaly, Failure to Thrive, Bone disease	Gaucher	c.1448T > C (p.(Leu483Pro))	Not detected but biochemically confirmed
Splenomegaly and anaemia	Gaucher	c.1448T > C (p.(Leu483Pro))	c.1504C > T (p.(Arg502Cys))
Hepatosplenomegaly, Growth retardation	Gaucher	c.1504C > T (p.(Arg502Cys))	c.721 G > A (p.(Gly241Arg))
Hepatosplenomegaly, Failure to thrive, anaemia	Gaucher	c.1504C > T (p.(Arg502Cys))	Not detected but biochemically confirmed
Splenomegaly, Failure to Thrive	Gaucher	c.754T > A (Phe252Ile))	c.1448T > C (p.(Leu483Pro))
Hepatosplenomegaly, Failure to thrive, anaemia	Gaucher	c.1504C > T (p.(Arg502Cys))	Not detected but biochemically confirmed
Hepatosplenomegaly, Anaemia, febrile neutropenia	Gaucher	c.259C > T (p.(Arg87Trp))	Not detected but biochemically confirmed
Hepatosplenomegaly, pancytopenia, poor wound healing	Gaucher		Not detected but biochemically confirmed
Hypertonia, Splenomegaly, Failure to thrive	Gaucher	c.1448T > C (p.(Leu483Pro))	Not detected but biochemically confirmed
Dysmorphic features, skeletal abnormalities, failure to thrive, corneal clouding	MPS I	c.589+2T > C	Not detected but biochemically confirmed
Dysmorphic features, hepatomegaly, abnormal teeth, Persistent umbilical hernia	MPS I	c.312dela (p.(Arg105Glyfs*3))	c.1162A > C (p.(Thr388Pro))
Dysmorphic features, deafness and speech delay, corneal clouding, short stature	MPS I	c.546 G > C (p.(Glu182Asp))	Not detected but biochemically confirmed
Dysmorphic features, deafness, skeletal abnormalities, hepatosplenomegaly	MPS II	c.263 G > C (p.(Arg88Pro))	
Dysmorphic features, developmental delay, teeth abnormalities, hepatosplenomegaly	MPS II	c.1010 G > A (p.(Trp337*))	
Dysmorphic features, deafness, skeletal abnormalities, developmental delay, positive family history	MPS II	c.257C > T (p.(Pro86Leu))	
Dysmorphic features, deafness, skeletal abnormalities, developmental delay, positive family history	MPS II	c.1010 G > A (p.(Trp337*))	
Dysmorphic features, hepatosplenomegaly, abnormal teeth, Skeletal abnormalities and contractures	MPS II	c.632del (p.(Lys211Argfs*2))	
Dysmorphic features, persistent and recurrent umbilical hernia, abnormal teeth, developmental delay, cardiomegaly	MPS II	c.683C > T (p.(Pro228Leu))	
Abnormal gait, dysmorphic features, skeletal abnormalities, abnormal teeth	MPS IVa	c.230C > G (p.(Pro77Arg))	c.245C > T (p.(Ser82Leu))

## Discussion

### Training

Training presented at the CHM was well received and rated as effective by all the participating doctors, with positive feedback for all the sections of the course. The participating doctors suggested a hybrid learning option for future training sessions. The COVID-19 pandemic has made it clear that in-person meetings, although still mostly preferable, are not the only way to present training. Online training could be performed at a time and pace that suits the clinician. If this approach is combined with personal case discussion, it could be the ideal way of empowering clinicians in Africa to better understand and diagnose rare diseases. Knowledge obtained from the in-person meeting also led to expansion of the network to other clinicians; this emphasizes the importance of training to empower clinicians to be able to train other clinicians in their workplace.

### Diagnostics

Most LSDs are caused by a deficiency of a specific lysosomal protein involved in lysosomal biogenesis. Except for mucopolysaccharidosis (MPS) type II and Fabry disease, which are inherited in an X-linked recessive manner, nearly all LSDs show autosomal recessive inheritance. Various reports regarding the prevalence of LSDs have been compiled but have focused only on selected populations. These studies have focused mainly on Middle Eastern, European, Asian and American populations, although in general, they have not been comprehensive enough to cover all LSDs. [9–18] Studies on African populations, on the other hand, have not received much consideration.

Over a period of approximately 2 years (April 2022–June 2024), a total of 56 samples were registered for this project. The most requested and most diagnosed LSDs were Gaucher and MPS. Most of the Gaucher diagnoses came from Tanzania, followed by Kenya, Sudan and Botswana. Most of the MPS diagnoses were from Kenya, followed by Tanzania, Nigeria, Sudan and Mozambique. The participants with a positive Gaucher diagnosis were mostly female, whereas the participants diagnosed with MPS were mostly male. The single Fabry diagnosis was for a male participant.

Most of the positive LSD results came from requests for that specific LSD. All the countries that have referred samples include samples that have shown a normal result. Although collectively common, individual rare disorders have a low prevalence. In this pilot study, the diagnostic yield from a limited set of targeted tests for rare diseases was notably high. This outcome is attributed primarily to the strategy of prioritizing patients with pathognomonic clinical features for initial testing. It is anticipated that subsequent cohorts, which may include patients with less specific phenotypes, will yield lower diagnostic rates. For these patients, whole-genome sequencing (WGS) or whole-exome sequencing (WES) should be considered first-line diagnostic approaches following multiple inconclusive screening attempts. Given the low prevalence of individual rare disorders, clinicians may often become discouraged and discontinue the diagnostic process when an initial diagnosis is not readily achieved. However, substantial phenotypic and genotypic overlap exists among rare diseases, underscoring the importance of persistence in the diagnostic workup. A comprehensive understanding of the most prevalent rare disorders in specific populations is necessary to inform the development of geographically appropriate diagnostic strategies. A significant challenge in sub-Saharan Africa (SSA) is the limited availability of diagnostic infrastructure, which has led to an underrepresentation of African phenotypic variability in current disease descriptions. Existing clinical characterizations are predominantly derived from European populations, potentially reducing diagnostic accuracy in African contexts.

In terms of artefactual results, the artefactual result from Sudan was the overlay of blood spots on the DBS card. This was the first sample received; thereafter, 2 Gaucher and 1 MPS I diagnosis were made. The other artefacts were from Tanzania and Mozambique; both countries are located on the eastern coast of the African continent and are known for their tropical, humid climates. Both Tanzania and Mozambique experienced above-average rainfall at the time these samples were collected. Studies have shown that LSD enzyme activity in blood samples prepared on DBS cards is very sensitive to temperature and humidity during sample collection, storage and transport [19–21]. These factors will be considered for future samples from these regions; we will expand on this in the strengths and limitations section.

When considering the efficiency of transporting samples across political borders and over vast distances via the established pathology network, the following was noted: Most samples reached the CHM in SA within 1–2 weeks. When considering the samples individually, we can see that the increased lead times in Kenya and Nigeria were due to a few outliers. Most of the samples reached the laboratory within 2 weeks. There were significant challenges for samples from Sudan to reach the CHM in SA. Sudan exhibited prolonged turnaround times due to the absence of integrated pathology networks and dependence on private couriers. Sample transport from Sudan to South Africa had no complications; however, problems with import permits and delays in the release of samples from the port were major pitfalls. Unfortunately, samples were referred only from Sudan until March 2023 (*n* = 5), before the civil war broke out in April 2023. The referring physician has subsequently not been able to refer any other samples since all courier services had been disrupted.

There was no correlation between the diagnostic result and the number of days until the sample was registered; i.e., increased time between sample collection and registration at the laboratory did not result in an increased number of artefactual results, thus DBS cards proved suitable for cross-border diagnostics and maintained sufficient stability for enzyme and genetic analyses.

### Clinical presentations and genetic variants

The request for initial testing resembles the classical features of the diseases that have been confirmed. For patients with Gaucher disease, splenomegaly with or without hepatomegaly and some haematological features were the main triggers. Requests for testing for MPS disorders were triggered by dysmorphic features, developmental delay, dental abnormalities and skeletal abnormalities. There is still a very low diagnostic yield for Fabry disease, which cannot be easily explained. The only confirmed case was from family cascade screening, and these patients are potentially adults with renal failure, early-onset stroke and cardiomyopathy; therefore, a more focused pilot program is needed.

Most genetic variants have been reported previously in at least one allele. In the cases where only one allele was found, the diagnosis was confirmed by biochemical measures, confirming that the phenotype was not novel.

For Gaucher disease, c.1448T>C (p.Leu483Pro), which is a variant that has been detected in India with visceral disease and late-onset neurological features, was the most common variant. In our context, they seem to represent a very severe early-onset disease with primarily visceral features. The second most frequent mutation observed was c. 1504C > T (p.Arg502Cys)), which is associated with nonneurological disease in most cases.

For MPS disorders, previously reported variants have been identified, but no specific pattern or common variants were identified in this small pilot study.

However, MPS II was the most prevalent MPS disorder, which is very similar to the picture in the Eastern Hemisphere rather than MPS1, which is more prevalent in other parts of the world.

### Strengths and limitations

The strengths of this study include the effective training of physicians as well as the functionality of pathology laboratory coverage in Africa. Training provided by the CHM was highly rated by the attending physicians, and the effectiveness was indicated by the high frequency of samples that tested positive for an LSD. Compared with the global incidence, the diagnostic hit rate for MPS, Gaucher and Fabry in this patient population (*n* = 56) is substantially greater, confirming the benefit of the training sessions hosted at CHM as well as the practicality of DBS cards as an effective alternative for genetic testing. DBS offers several logistical advantages for remote health and surveillance programs, particularly for screening and surveying hard-to-reach populations. For many of these tests, highly sensitive biomarker analysis is important for reducing the risk of missed positive cases. Analytical sensitivity includes not only the performance of a downstream platform but also the preanalytical workflow that starts with the collection of a sample from an individual [7].

Compared with Sudan, which does not fall within the established pathology laboratory network, the other countries had shorter lead times to reach the South African laboratory because of the established pathology laboratory coverage and infrastructure it provides to these countries.

Some limitations were identified. First, genetic testing was only performed for enzyme-positive samples; enzyme-negative, inconclusive or artefactual samples were not genetically assessed, which may have led to missed diagnoses and an underestimation of true prevalence. Future project phases will incorporate molecular evaluation for enzyme-negative and artefactual samples to strengthen diagnostic validity.

Second, the small, unevenly distributed cohort (*n* = 56) and reliance on clinician-driven recruitment likely introduced both representativeness and ascertainment bias. It should however be noted that, given the inherently low prevalence of rare diseases, a large patient cohort was not anticipated, unlike studies involving more common conditions. Additionally, logistical disparities between countries with established laboratory transport networks and those dependent on private couriers further limited regional comparability, with the latter additionally affected by civil-war-related disruptions, all of which must be considered when interpreting the findings.

The greatest identified limitation of this study is the number of artefactual results from humid, tropical regions. Further investigations (e.g., the inclusion of humidity indicator cards to accompany the collection kits) are needed to confirm that humidity and temperature are the causes of the artefactual results. To limit this going forward, clinicians will be asked to store and transport samples with additional desiccant packs as well as additional cooling measures such as gel packs during periods of high humidity and/or temperatures.

Finally, training assessment relied solely on programme attendance and case-study completion, which could be strengthened to better evaluate learning outcomes. Future iterations will incorporate pre/post testing, objective competency assessments, and longitudinal monitoring of referral behaviour to more accurately measure training effectiveness.

### Future actions

To further advance the pilot project, preliminary results and key outcomes will be presented at an upcoming rare disease symposium or conference, with a preference for an African-based event to increase regional engagement. Additionally, monthly catch-up calls will be established with clinicians to facilitate ongoing discussions, address challenges, and refine the study approach. To complement these efforts, a dedicated WhatsApp group will be created as part of the pilot project, enabling continuous communication and collaboration among clinicians, thereby fostering real-time knowledge exchange and streamlined coordination.

## Conclusion

This study highlights the effectiveness of clinician training, DBS as stable and essential tool for diagnostic testing in Africa, the feasibility of diagnostic sample referrals across multiple African countries, and the potential impact of expanding access to rare disease diagnostics in underserved regions. However, challenges remain, particularly regarding sample integrity in tropical climates, referral gaps from certain countries, and logistical delays influenced by pathology network coverage. Addressing these barriers will be critical for optimizing diagnostic capacity. Moving forward, ongoing engagement with clinicians through structured follow-ups and digital communication platforms will strengthen collaboration and improve diagnostic outcomes. Presenting findings at an African rare disease symposium will further contribute to regional awareness and capacity building in rare disease diagnostics.

## Electronic supplementary material

Below is the link to the electronic supplementary material.

Supplementary material 1

## Data Availability

The raw research data could not be shared due to ethical restrictions.

## Abbreviation

ABG: Acid beta-glucosidase (beta-glucocerebrosidase)
ASM: Acid sphingomyelinase
ASMD: Acid sphingomyelinase deficiency
CDC: Centers for Disease Control and Prevention
CHM: Centre for Human Metabolomics
DBS: Dried blood spot
ESI: Electrospray ionization
GAA: Acid alpha-glucosidase
GALC: Galactocerebrosidase
GLA: Alpha-galactosidase
HIV: Human Immunodeficiency Virus
HREC: Human Research Ethics Committee
IGA: International Gaucher Alliance
IMD: Inherited metabolic disease
IQR: Interquartile range
LC-MS/MS: Liquid chromatography tandem mass spectrometry
LSD: Lysosomal storage disorder
MSMS: Tandem mass spectrometry (multiple-stage mass spectrometry)
MPS: Mucopolysaccharidosis
NWU: North-West University
RD: Rare disease
SSA: Sub-Saharan Africa
TB: Tuberculosis
TQ: Triple quadrupole
WES: Whole-exome sequencing
WGS: Whole-genome sequencing
