# Supplementary figures and images for: Improving access to rare disease diagnostics in Africa: insights from a multinational pilot study
Source: Orphanet J Rare Dis. 2026 Feb 5;21:44. doi: 10.1186/s13023-026-04202-y (PMC12879359; doi:10.1186/s13023-026-04202-y)

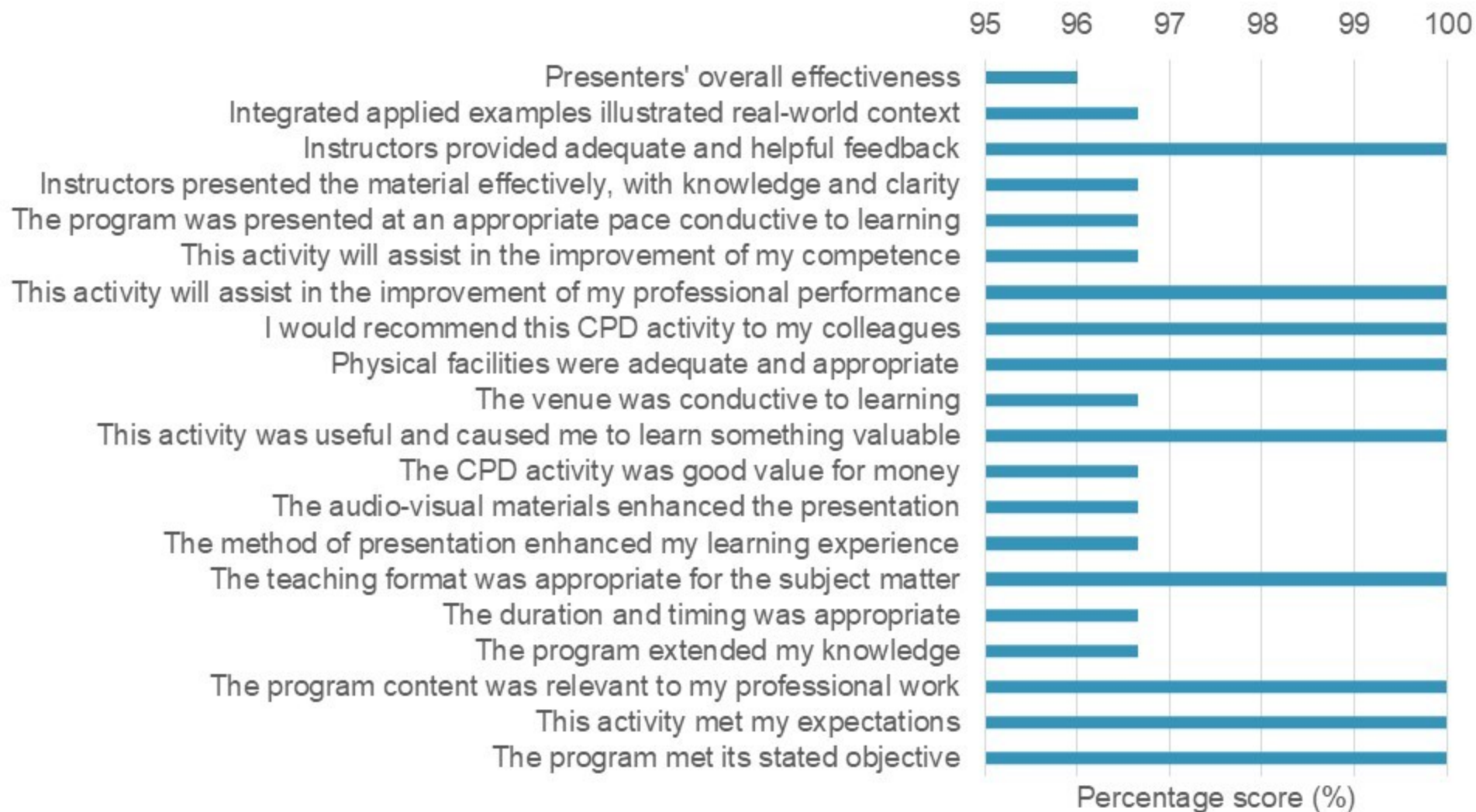

Supplement: Supplementary file 1 — Supplementary material 1 [file 13023_2026_4202_MOESM1_ESM.pdf]
